# Targeting of topoisomerases for prognosis and drug resistance in ovarian cancer

**DOI:** 10.1186/s13048-016-0244-9

**Published:** 2016-06-18

**Authors:** Yang Bai, Liang-Dong Li, Jun Li, Xin Lu

**Affiliations:** Obstetrics and Gynecology Hospital, Fudan University, Shanghai, 200011 China; Department of Obstetrics and Gynecology of Shanghai Medical College, Fudan University, Shanghai, 200032 China; Shanghai Key Laboratory of Female Reproductive Endocrine Related Diseases, Shanghai, 200011 China; Department of Breast Surgery, Key Laboratory of Breast Cancer in Shanghai, Fudan University Shanghai Cancer Center, Shanghai, 200030 China; Department of Oncology, Shanghai Medical College, Fudan University, Shanghai, 200030 China; Permanent address: Department of Gynecology, Obstetrics and Gynecology Hospital of Fudan University, No.419, Fangxie Road, Shanghai, 200011 China

**Keywords:** Prognosis, Epithelial ovarian cancer (EOC), Topoisomerases, Kaplan Meier plotter, Oncomine

## Abstract

**Backgroud:**

As magicians of the DNA world, topoisomerases resolve all of the topological problems in relation to DNA during a variety of genetic processes. While the prognostic value of topoisomerase isoenzymes in epithelial ovarian carcinoma (EOC) is still elusive. In current study, we investigated the prognostic value of topoisomerase isoenzymes in the EOC patients. Kaplan Meier plotter (KM plotter) database were used to assess the relevance of individual topoisomerase isoenzyme mRNA expression to EOC patients overall survival (OS), in which updated survival information and gene expression data were from a total of 1,648 EOC patients.

**Results:**

High expression of TOP1 and TOP2A were found to be correlated to worse OS in all patients and serous patients, but not in endometrioid patients. Contrary to TOP1 and TOP2A, TOP3A and TOP3B expression were associated with better OS in all patients and serous patients, but not in endometrioid patients. While TOP2B were not found any significant prognostic value for EOC patients. From the Oncomine database, we also found widespread upregulation in the expression of TOP1 and TOP2A genes in primary tumor tissues. Albeit limited in number, all datasets exhibiting differential expression showed TOP3A and TOP3B under-regulated.

**Conclusion:**

These results strongly supported that TOP1 and TOP2A were potential biomarkers for predicting poor survival of EOC patients, while TOP3A and TOP3B were expected to be further exploited as tumor suppressors. Comprehensive understanding of the topoisomerase isoforms may have guiding significance for the diagnosis treatment and prognosis in EOC patients.

**Electronic supplementary material:**

The online version of this article (doi:10.1186/s13048-016-0244-9) contains supplementary material, which is available to authorized users.

## Backgroud

Epithelial ovarian carcinoma (EOC) is one of the most lethal gynecological malignancies and is usually diagnosed at an advanced stage with a low 5-year survival rate of 25–30 %. Surgery for staging and optimal cytoreduction followed by adjuvant chemotherapy with platinum/paclitaxel combination is the standard treatment guideline, which gets an achievement of high response rate [1]. Although patients with EOC generally respond to initial platinum-based chemotherapy, almost 70 % of advanced stage patients will develop recurrent cancer, [2] which is determined by the extent of residual tumor at primary surgery and sensitivity to platinum-based therapy Hence, there is a need to take into account the use of second-line chemotherapeutic options for EOC patients, such as retreatment with paclitaxel and carboplatin, as well as treatment with pegylated liposomal doxorubicin (PLD), docetaxel, or gemcitabine, topotecan, and avastin, with a response rate between 10 % and 30 % [3, 4].

DNA topoisomerases have been described as the targets of important anticancer agents, such as TOP1 targeted drugs-topotecan and irinotecan, TOP2 targeted drugs-etoposide and doxorubicin etc. [5, 6] In addition, alterations of topoisomerase enzymatic activity can also lead to atypical multidrug resistance [7, 8].

The critical actions of DNA topoisomerases include DNA strand separation for transcription and replication, the flawless segregation of two identical copies of entire genomes following replication, and the formidable genomic compaction in cells. DNA topoisomerases are classified as type I and type II, and the two types can be further divided into four subfamilies: IA, IB, IIA and IIB [9, 10]. The human genome encodes six topoisomerases, which include type IA, IB and IIA [11, 12]. Each of the subfamilies IA (TOP3A and TOP3B), IB (TOP1 and mitochondrial TOP1) and IIA (TOP2A and TOP2B), are known for two enzymes [13, 14].

For type I enzymes, they transiently break the single DNA strand at a time; on the contrary, for type II enzymes, as a dimeric enzyme molecule, they transiently break a pair of strands in a DNA double helix in concert. In contrast to the type IA and IB DNA topoisomerases, the type IIA and IIB enzymes catalyze the ATP dependent traverse of one intact DNA duplex through another [15, 16].

Type IA (TOP3A and TOP3B) enzymes are the only enzymes that relax negative but not positive supercoiling. It resolves recombination intermediates and plays a role as decatenase on nicked DNA during their replication. There is strong evidence that they physically interacts with SGS1 helicase, [17, 18] as well as other members of the RecQ family, [19, 20] indicating a function in maintaining genomic stability.

Type IB (TOP1) and type IIA (TOP2A and TOP2B) enzymes mainly function to relax DNA to remove negative and positive supercoils during replication and transcription. In eukaryotes, coiling a DNA into a compact form can be implemented by either a type IB or a type II enzyme [10]. TOP2A is tightly linked to cell multiplication, that is, its expression increases 2 to 3 fold during G2/M and is orders of magnitude higher in rapidly proliferating than in non-dividing cells. Whereas TOP2B is also expressed in quiescent cells [5].

In EOC, TOP1 and TOP2A expression was observed in a large proportion of cases (30–70 %) [21]. While, there is limited data about the prognostic value of topoisomerase expression in ovarian cancer, especially of the TOP3A and TOP3B. And a significant correlation between elevated topoisomerase expression and EOC sensitivity to multidrug is still controversial. In this study, we aimed at identifying the role of DNA topoisomerase isoforms expression in the prognosis of EOC, which may help to assess the patients’ risk profile and facilite the development of more effective therapeutic strategies for EOC patients.

## Methods

### The Kaplan Meier plotter

The Kaplan Meier plotter (KM plotter) was capable to assess the effect of 54,675/22,277 genes on survival using 10,188 tumor samples, which included 1648 ovarian, 4142 breast, 1065 gastric and 2437 lung cancer patients with a mean follow-up of 69/40/49/33 months (http://kmplot.com/analysis/). The background database was established by the use of gene expression and patients survival information from the Gene Expression Omnibus (GEO, http://www.ncbi.nlm.nih.gov/geo/), Cancer Biomedical Informatics Grid (caBIG, http://ncip.nci.nih.gov), European genome phenome archive (EGA, https://www.ebi.ac.uk/ega/), and the Cancer Genome Atlas (TCGA, http://cancergenome.nih.gov), which was handled by a MySQL server and integrates clinical data and gene expression information simultaneously [22–24].

With the purpose to analyze a particular gene’s prognostic value, the cohorts were divided into two groups according to the median (or upper/lower quartile) expression of the gene. The overall survival (OS), relapse free survival (RFS), etc. could be compared between the two groups [25]. Briefly, the five genes (TOP1A, TOP2A, TOP2B, TOP3A, and TOP3B) were entered into the database (http://kmplot.com/analysis/) respectively to obtain the Kaplan-Meier survival plots in which the number at risk was indicated below the main plot. Log rank *P* value and hazard ratio (and 95 % confidence intervals) were calculated and displayed on the webpage.

### Oncomine analysis

Oncomine was a cancer microarray database and web-based data mining platform aimed at facilitating new discovery from genome-wide expression analyses, in which exploration for differential expression analyses comparing most major types of cancer with respective normal tissues as well as clinical-based and pathology-based analyses were available [26].

The individual gene expression level of TOP1A, TOP2A, TOP2B, TOP3A, and TOP3B was analyzed using Oncomine. We compared mRNA levels of cancer vs. normal patient datasets. In order to reduce false discovery rate, we selected 2.0 fold change, *P* value = 0.01, and Top 10 % as threshold.

## Results

Among all the six topoisomerase genes, mitochondrial TOP I was not found in www.kmplot.com, maybe on account of its peculiarity from the nuclear genome and we concluded the other five topoisomerase genes in this article.

**Fig. 1:**
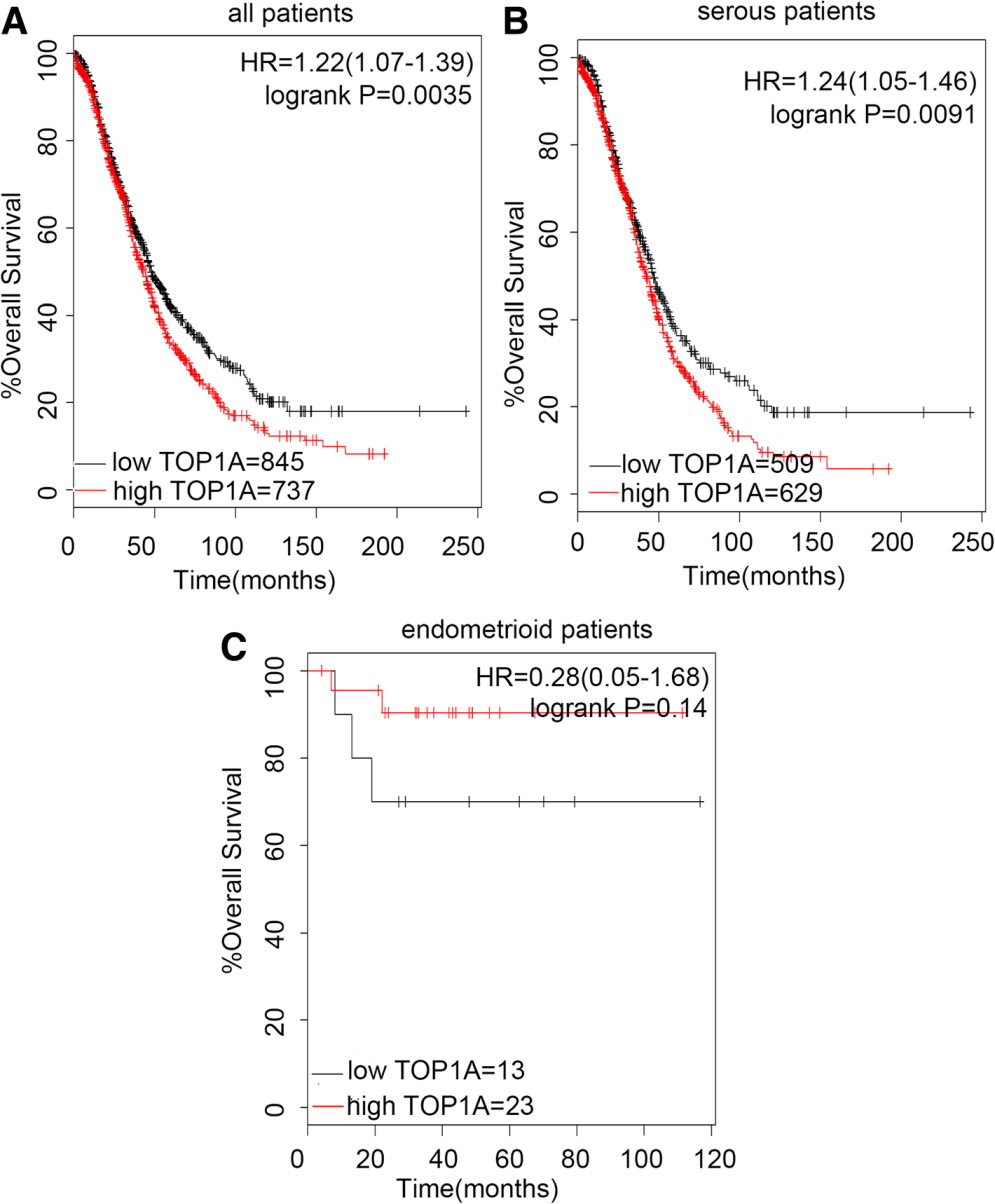
The prognostic value of TOP1A expression. Notes: The desired Affymetrix ID is valid: 208900_s_at (TOP1A). **a**. Survival curves are plotted for all patients (*n* = 1582). **b**. Survival curves are plotted for serous patients (*n* = 1138). **c**. Survival curves are plotted for endometrioid patients (*n* = 36). Data was analyzed using Kaplan Meier Plotter (www.kmplot.com). Abbreviation: HR, hazard ratio; CI, confidence interval

First, we examined the prognostic value of the expression of TOP1 in www.kmplot.com. The desired Affymetrix was valid: 208900_s_at (TOP1). Survival curves were plotted for all patients (*n* = 1,582), for serous (*n* = 1138), and for the endometrioid (*n* = 36). It was found that TOP1 mRNA high expression was correlated to significantly worse OS for all EOC patients followed for 20 years, (HR 1.22 [1.07–1.39], *P* = 0.0035) (Fig. 1a). In addition, TOP1 mRNA high expression was also found to be correlated to significantly worse OS in serous patients, (HR 1.24 [1.05–1.46], *P* = 0.0091) (Fig. 1b), but insignificantly better OS in endometrioid patients, (HR 0.28 [0.05–1.68], *P* = 0.14) (Fig. 1c).

**Fig. 2:**
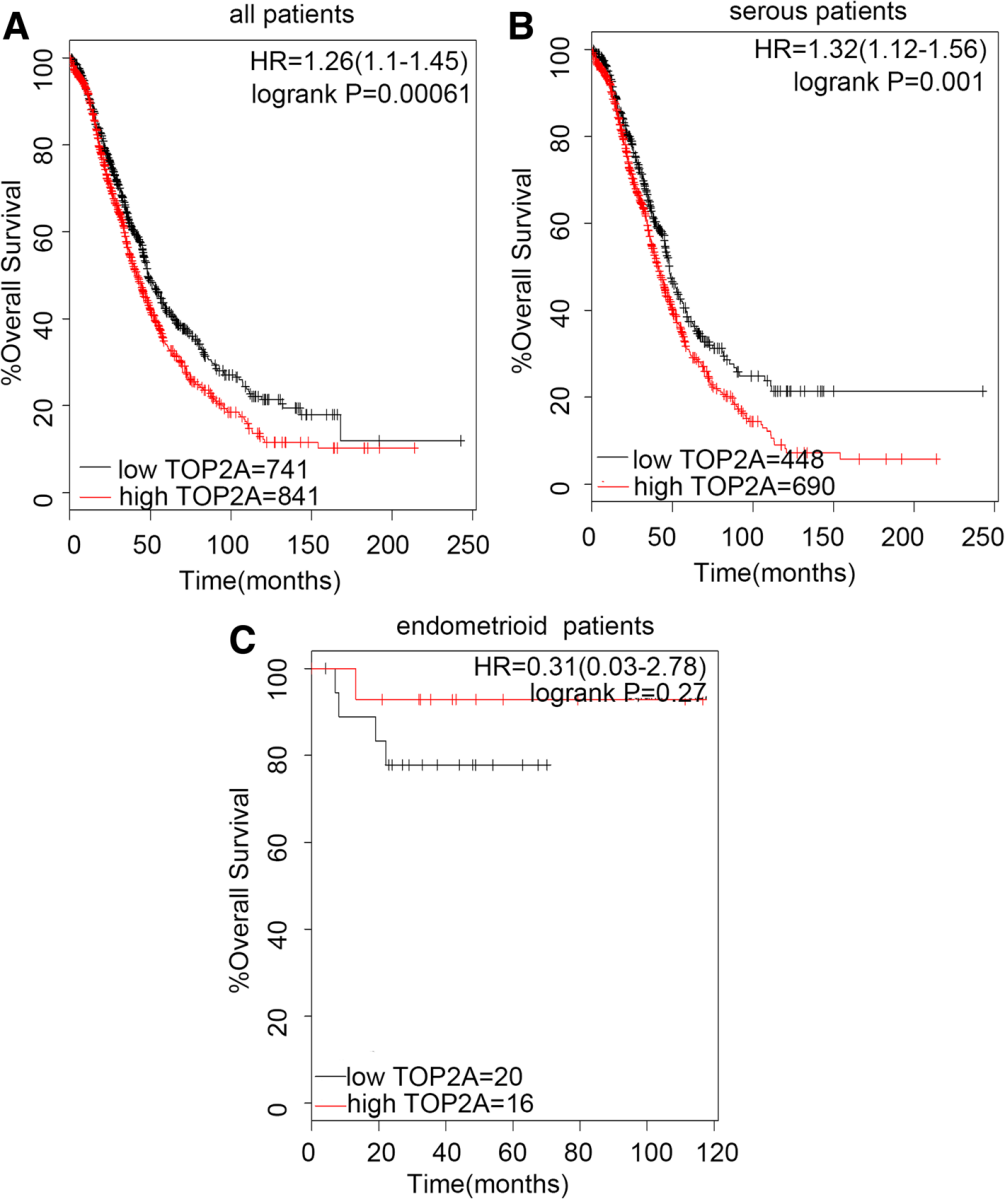
The prognostic value of TOP2A expression. Notes: The desired Affymetrix ID is valid: 201291_s_at (TOP2A). **a**. Survival curves are plotted for all patients (*n* = 1582). **b**. Survival curves are plotted for serous patients (*n* = 1138). **c**. Survival curves are plotted for endometrioid patients (*n* = 36). Data was analyzed using Kaplan Meier Plotter (www.kmplot.com). Abbreviation: HR, hazard ratio; CI, confidence interval

Then we determined the prognostic effect of the expression of TOP2A in www.kmplot.com. The desired Affymetrix was valid: 201291_s_at (TOP2A). TOP2A mRNA high expression was also found to be correlated to significantly worse OS in all patients (*n* = 1582), (HR 1.26 [1.1–1.45], *P* = 0.00061) (Fig. 2a), and in serous patients (*n* = 1138), (HR 1.32 [1.12–1.56], *P* = 0.001) (Fig. 2b), but insignificantly better OS in endometrioid patients (*n* = 36), (HR 0.31 [0.03–2.78], *P* = 0.27) (Fig. 2c).

**Fig. 3:**
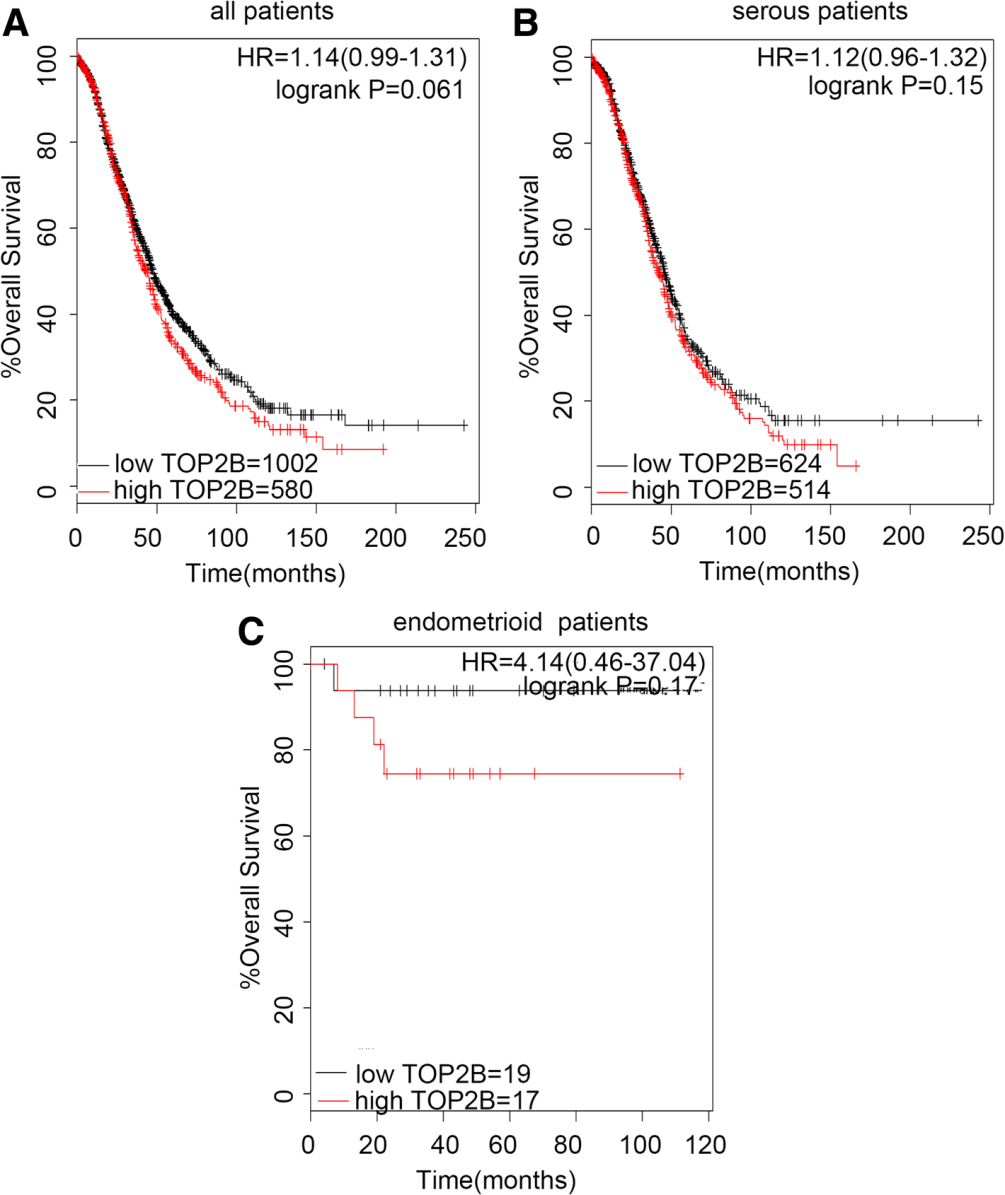
The prognostic value of TOP2B expression. Notes: The desired Affymetrix ID is valid: 211987_at (TOP2B). **a**. Survival curves are plotted for all patients (*n* = 1582). **b**. Survival curves are plotted for serous patients (*n* = 1138). **c**. Survival curves are plotted for endometrioid patients (*n* = 36). Data was analyzed using Kaplan Meier Plotter (www.kmplot.com). Abbreviation: HR, hazard ratio; CI, confidence interval

Figure 3 showed the prognostic effect of the expression of TOP2B in www.kmplot.com. The desired Affymetrix IDs was valid: 211987_at (TOP2B). However, the curves showed that TOP2B expression above or below the median in all EOC patients did not separate them into significantly different prognostic groups, (*n* = 1582), (HR 1.14 [0.99–1.31], *P* = 0.061) (Fig. 3a). In addition, the curves also did not show any differences among the serous patients, (*n* = 1138), (HR 1.12 [0.96–1.32], *P* = 0.15) (Fig. 3b), while, for endometrioid patients, albeit separated into different groups, not significantly (*n* = 36), (HR 4.14 [0.46–37.04], *P* = 0.17) (Fig. 3c).

**Fig. 4:**
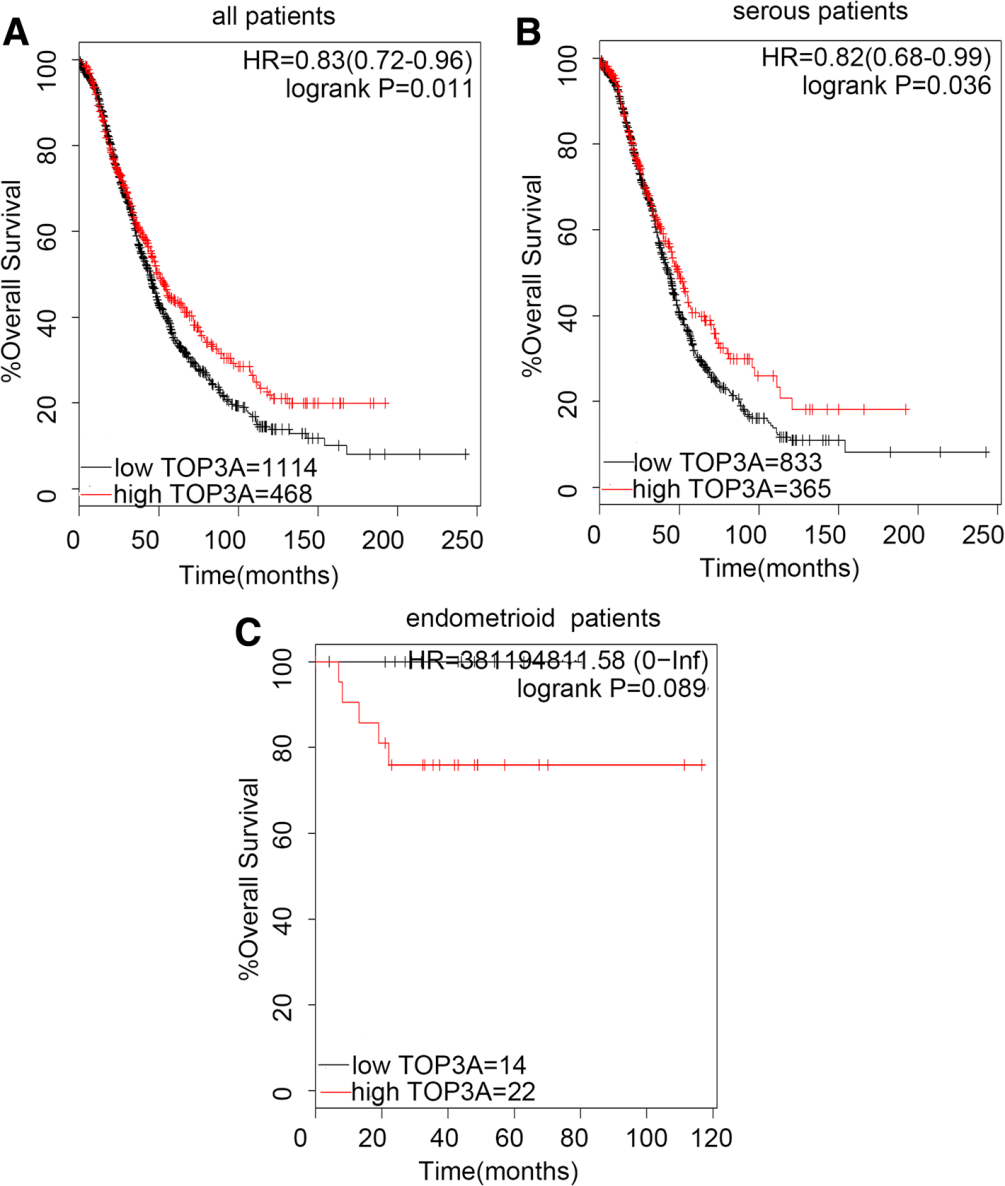
The prognostic value of TOP3A expression. Notes: The desired Affymetrix ID is valid: 204946_s_at (TOP3A). **a**. Survival curves are plotted for all patients (*n* = 1582). **b**. Survival curves are plotted forserous patients (*n* = 1138). **c**. Survival curves are plotted for endometrioid patients (*n* = 36). Data was analyzed using Kaplan Meier Plotter (http://kmplot.com/analysis/). Abbreviation: HR, hazard ratio; CI, confidence interval

Interestingly, TOP3A and TOP3B expression showed favorable prognostic value in www.kmplot.com. The desired Affymetrix IDs was valid: 204946_s_at (TOP3A). The high expression of TOP3A was found to be correlated to significantly better OS for all EOC patients, (*n* = 1582), (HR 0.83 [0.72–0.96], *P* = 0.011) (Fig. 4a), and for serous patients, (*n* = 1138), (HR 0.83 [0.68–0.99], *P* = 0.036) (Fig. 4b), but insignificantly worse OS for endometrioid patients, (*n* = 36), (HR 381194811.58 [0 -lnf], *P* = 0.089) (Fig. 4c).

**Fig. 5:**
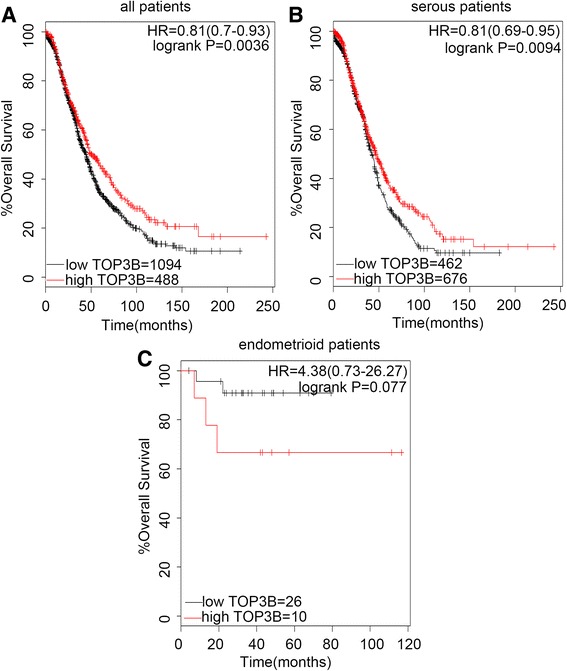
The prognostic value of TOP3B expression. Notes: The desired Affymetrix ID is valid: 215781_s_at (TOP3B). **a**. Survival curves are plotted for all patients (*n* = 1582). **b**. Survival curves are plotted for serous patients (*n* = 1138). **c**. Survival curves are plotted for endometrioid patients (*n* = 36). Data was analyzed using Kaplan Meier Plotter (www.kmplot.com). Abbreviation: HR, hazard ratio; CI, confidence interval

Next, the prognostic value of TOP3B in EOC patents was shown in Fig. 5. The desired Affymetrix IDs was valid: 215781_s_at (TOP3B). The TOP3B high expression was associated with the significantly better OS in all EOC patients, (*n* = 1582), (HR 0.81 [0.7–0.93], *P* = 0.0036) (Fig. 5a), and in serous patients, (*n* = 1138), (HR 0.81 [0.69–0.95], *P* = 0.0094) (Fig. 5b). While for endometrioid patients, it was related to insignificantly worse prognosis, (*n* = 36), (HR 4.38 [0.73–26.27], *P* = 0.077) (Fig. 5c).

We also sought to explore the relationship between topoisomerases expression and the clinicopathological features for the EOC patients, such as grades, clinical stages, and TP53 mutation. As was shown in Table 1, both of the TOP1 and TOP2A were found to be associated with significantly worse OS in all grade III EOC patients, nevertheless, both of the TOP3A and TOP3B were correlated to significantly better OS in all grade III EOC patients. From Table 2, both TOP2A and TOP2B were found to be correlated to significantly shorter OS in all stage I EOC patients and TOP1 was found to be associated with insignificantly worse OS, while the expression of TOP3A and TOP3B indicated significantly better OS in all stage III EOC patients. In EOC patients, negtive correlations between OS and TOP2A mRNA expression companied by TP53 mutation or TOP1 mRNA expression with wild TP53were revealed. Although insignificant OS for patients by other genes, individually they showed opposite prognostic value between TP53 wild type or mutation type patients, implying the correlation between topo members and TP53 (Table 3)

**Table 1:** Correlation of topoisomerase isoenzymes with tumor grade of all EOC patients

Topoisomerases	Grades	Case-low	Case-high	HR (% 95 CI)	*P*-value
TOP1	I	20	38	2.07 (0.59 − 7.28)	0.25
	II	105	210	1.29 (0.9 − 1.85)	0.16
	III	405	563	1.3 (1.09 − 1.55)	0.0033
TOP2A	I	38	18	2.79 (1.04 − 7.47)	0.033
	II	92	223	1.46 (1.07 − 1.98)	0.016
	III	275	693	1.21 (1.02 − 1.44)	0.028
TOP2B	I	34	22	0.51 (0.18 − 1.41)	0.18
	II	200	115	1.48 (1.08 − 2.02)	0.014
	III	249	719	1.12 (0.92 − 1.36)	0.27
TOP3A	I	36	20	0.48 (0.17 − 1.37)	0.16
	II	103	212	0.79 (0.58 − 1.09)	0.16
	III	670	298	0.8 (0.66 − 0.97)	0.021
TOP3B	I	41	15	0.48 (0.14 − 1.69)	0.25
	II	137	178	1.25 (0.91 − 1.7)	0.16
	III	698	270	0.73 (0.6 − 0.88)	0.0012

*Abbreviation*: *HR* hazard ratio; *CI* confidence interval

**Table 2:** Correlation of topoisomerase isoenzymes with clinical stage of all EOC patients

Topoisomerases	Clinical stages	Case-low	Case-high	HR (%95 CI)	*P*-value
TOP1	I	19	55	4.47 (0.57 − 34.8)	0.12
	II	43	16	2.54 (0.81 − 7.91)	0.096
	III	718	264	1.18 (0.97 − 1.42)	0.096
	IV	69	97	1.17 (0.8 − 1.71)	0.43
TOP2A	I	37	37	10.37 (3.04−35.44)	5.8e − 06
	II	25	34	2.04 (0.68 − 6.11)	0.19
	III	277	705	1.21 (1−1.46)	0.053
	IV	74	92	0.76 (0.52 − 1.13)	0.18
TOP2B	I	34	40	4.64 (1.02 − 21.21)	0.029
	II	24	35	0.29 (0.09 − 0.94)	0.028
	III	249	719	0.87 (0.72 − 1.06)	0.18
	IV	94	72	1.4 (0.96 − 2.06)	0.082
TOP3A	I	54	20	1.84 (0.58 − 5.8)	0.29
	II	42	17	0.44 (0.1 − 1.98)	0.27
	III	716	266	0.7 (0.57 − 0.86)	0.00045
	IV	59	107	0.74 (0.5 − 1.09)	0.13
TOP3B	I	42	32	3.05 (0.91 − 10.2)	0.057
	II	35	24	1.76 (0.58 − 5.33)	0.31
	III	675	307	0.75 (0.63 − 0.91)	0.003
	IV	64	102	0.73 (0.5 − 1.08)	0.11

*Abbreviation: HR* hazard ratio; *CI* confidence interval

**Table 3:** Correlation of topoisomerase isoenzymes with TP53 status of all EOC patients

Topoisomerases	TP53 status	Case-low	Case-high	HR (% 95 CI)	*P*-value
TOP1	Mutated	109	303	0.83 (0.63−1.09)	0.18
	Wild type	54	32	1.91 (1.04−3.49)	0.033
TOP2A	Mutated	278	161	1.32 (1.02−1.7)	0.036
	Wild type	30	56	0.74 (0.41−1.33)	0.31
TOP2B	Mutated	252	187	0.78 (0.6−1.02)	0.066
	Wild type	38	48	1.34 (0.73−2.46)	0.34
TOP3A	Mutated	300	139	1.17 (0.89−1.52)	0.26
	Wild type	63	23	0.68 (0.33−1.42)	0.3
TOP3B	Mutated	304	135	1.26 (0.97−1.64)	0.086
	Wild type	42	44	1.45 (0.81−2.62)	0.21

.

Given that the expression level of the five genes had significant influence on patients’ survival, then we were interested in investigating their differential expression between normal and cancer tissues. Therefore, we extracted the summary data on transcript expression for the individual gene from the Oncomine database for most major tumors, focusing on datasets of clinical specimens of cancer vs. normal patients.

**Fig. 6:**
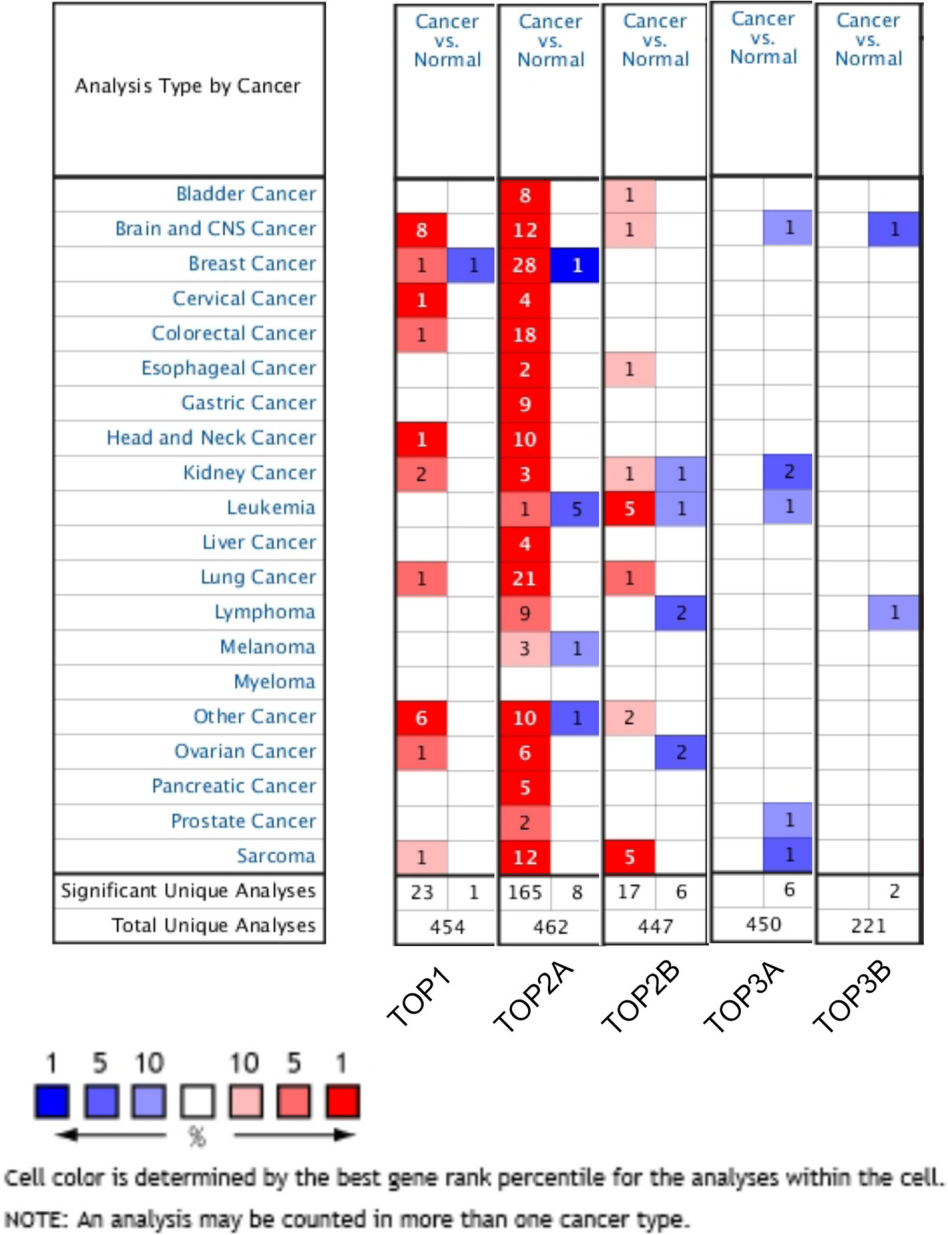
The individual gene expression level of TOP1A, TOP2A, TOP2B, TOP3A, and TOP3B in tumor vs. normal tissues. Notes: Various types of cancer were shown in the figure. And the expression level of six topoisomerase family genes were shown in cancer vs normal tissue. Data was analyzed using oncomine (www.oncomine.com). The datasets were obtained with the following parameters: *P* value threshold of 0.01, more than 2 fold change and top 10 % of the gene rank

As was shown in Fig. 6, for TOP1 (23 vs. 1), TOP2A (165 vs. 8) and TOP2B (17 vs. 6), overwhelming majority of the datasets with differential expression showed gene overexpression in tumor vs. normal tissues. For TOP3A (0 vs. 6) and TOP3B (0 vs. 2), although limited, all datasets with any expression changes, showed gene downregulation in tumor vs. normal tissues. It was accordant to the better OS for the TOP1, and TOP2A and worse OS for the TOP3A and Top3B.

## Discussion

By allowing DNA double helices or strands to pass through each other, DNA topoisomerases could solve all of the topological problems of deoxyribonucleic acid in replication, transcription and other cellular transactions. TOP1, TOP2A and TOP2B levels in several human cancers were reported to be higher than those of normal or benign tissues, [7, 21, 27, 28] which was in accordance to the results from Oncomine.

Numerous reports had demonstrated that the TOP1 gene copy number, mRNA and protein level, as well as enzyme activity were associated with unfavorable prognosis in oncotherapy [29–31]. Only individual data revealed the prognostic effect of topoisomerase expression in EOC patients, and our large-scale analysis that the negative correlation between the TOP1 and prognosis might have profound significance. Lee et.al revealed that the overexpression of TOP1 in EOC was correlated to the International Federation of Gynecologists and Obstetricians (FIGO) stages and was associated with advanced stage [30], which might reflect a high progressive growth of ovarian cancer. However, some other studies found that no obvious correlation was observed between the TOP1 expression and the staging and grading of ovarian cancer [32, 33]. In our current study, the high expression of TOP1 predicted insignificantly worse OS in clinical stage I and II patients and significantly worse OS in the pathological grade III patients.

Manipulating topoisomerase activity by poisoning or catalytic inhibition has been widely mined to kill cancer cells [5, 34, 35] Stabilization of topoisomerase cleavage complex (TOPcc) is one of the most reported and clinically important steps, of which by misaligning the DNA ends, preventing re-ligation, and thus trapping the enzyme on DNA, creating protein-linked DNA breaks (PDBs), eventually damaging DNA, inducing cell cycle arrest, even apoptotic cell death [36]. The enzymatic activities of topoisomerases can be interfered at various stages, such as binding to DNA or the combining and hydrolysis of ATP [37].

Several studies had indicated that TOP1 overexpression in human solid tumors maybe a potential predictive biomarker for TOP1 poisons [30, 32, 38, 39]. As topoisomerase I inhibitors, topotecan and irinotecan were FDA approved drugs used for the second-line chemotherapy of advanced or recurrent ovarian cancer patients due to refractory to first line drugs, e.g. paclitaxel plus platinum-based doublet chemotherapy. In our results, TOP1 expression above or below the median still significantly separated all patients, with chemotherapy process containing topoisomerase I inhibitor-topotecan, into different prognostic groups (Additional file 1: Table S1). The possible reasons might include: ① It was reported that in colorectal cancer the TOP1 inhibitor could induce autophagy and reduce apoptosis by activating the AMPK-TSC2-mTOR pathway [40]; Besides, topotecan could induce cytoprotective autophagy in wild-type TP53 colon cancer cells while not in TP53 mutanted or knockout cells [41]. ② Some mutations at G717V and T729I amino acid residues of TOP1 gene were identified to exert a synergetic effect on CPT resistance by targeting the catalytic site of the TOP1-DNA complexes [42]. ③ Zander et al. had identified that overexpression of Abcg2/Bcrp and significant reduced protein levels of the drug target TOP1 (without changing the levels of mRNA) maybe mechanisms of in vivo resistance [43]. ④ In patients, the subsequent single-agent chemotherapy with non-platinum drugs would lead to a short-lived response rates of around 20 % [2].

Topoisomerase IIα (TOP2A) and topoisomerase IIβ (TOP2B) were two different isoforms of type II topoisomerase, and they showed difference in biochemical, pharmacological, and physiological properties. So far, numerous studies had mainly focused on TOP2A. Elevated TOP2A mRNA were observed in high grade ovarian cancers as well as advanced stage diseases, and patients with overexpression of nuclear TOP2A protein had a marked decreased OS [44–46]. In our study, for all EOC patients and serous patients, the TOP2A overexpression was associated with significantly worse OS, but insignificantly better OS in endomitrioid patients, maybe due to tumor heterogeneity as well as the small and unbalanced sample sizes. For patients with whether low or high pathological grade, overexpression of TOP2A indicated a shorter OS, but its high expression in advanced stage patients is not associated with the significantly better or worse OS.

The role of TOP2B in tumor was still controversial. Being also expressed in quiescent cells in all tissues, the expression level of TOP2B was not changed during the cell cycle. [47] Few studies had focused on the correlation between the overexpression of TOP2B and cancer patients outcome. A study suggested that a type IIβ but rather the type IIα topoisomerase was more closely correlated with the development of secondary malignancy, treated by the type II topoisomerase targeting drugs, such as doxorubicin and etoposide [48]. Song et al. reported that the high expression level of the type IIβ topoisomerase was correlated with longer survival in acute myeloid leukaemia (AML) patients with M2 subtype [49]. Das et al. reported the increased exprtession of TOP2B was associated with the sensitivity of neuroblastoma cells to etoposide [50]. Another study discovered that the type IIβ protein levels correlated better with type II topoisomerase activity than type IIα protein levels and indicated type IIβ might be a target for chemotherapy in ovarian cancer [51]. While in our study, the high expression of TOP2B seemed irrelevant to the prognosis of all EOC patients. The exact role of TOP2B in tumor was expected to be elucidated.

The biologic role of TOP3 remained poorly understood, as well as the prognosis value of TOP3A and TOP3B in cancer patients. TOP3A was involved in DNA repair surveillance and cell-cycle checkpoints aimed at maintaining genomic stability possibly through formatting complex with BLM, RMI1, and RMI2 to form the BLM complex to dissolution of double Holliday junctions [52, 53]. It was reported that TOP3A interacted with TP53, regulating the expression of TP53 and P21, and contributed to the TP53-mediated tumor suppression [54]. TOP3B was the newest member of topoisomerase family, and played an important role in promoting transcription, preventing DNA damage, and reducing the frequency of chromosomal translocations [55]. It was suggested that topoisomerase IIIβ played an essential role in chromosome stability in normal cells, as well as in cancer cells [56]. Oliveira-Costa et al. [57] reported that high expression of TOP3B was correlated to shorter OS and metastasis in patients with invasive breast cancers. Different from above results, our results indicated that both the overexpression of TOP3A and TOP3B were associated with better OS in all EOC patients, as well as serous patients, the idea of which was put forward for the first time.

For the endometrioid patients, from Fig. 1 to Fig. 5, the curves showed that the topoisomerase mRNA above or below the median separated the cases into significantly different prognostic groups, usually opposite to the all patients and serous patients. While, maybe due to the small and unbalanced sample sizes, five of the *P* values were greater than 0.05. If the sample size was enlarged, the significant prognostic value of topoisomerase isoforms mRNA in endometrioid patients might be seen. This reflected the heterogeneity between different subtypes of one tumor.

On account of their differential structures and functions, the type IB (TOP1) or type II (TOP2A) enzymes are expected to be more suitable than type IA (TOP3A and TOP3B) enzymes for solving the topological problems that occurred during DNA chain elongation in replication [10]. Based on previous evidences as well as our results, TOP1 and TOP2A were strongly supported to potential biomarkers for predicting poor survival of EOC patients, while TOP3A and TOP3B were expected to be further exploited for their potential antitumor effects.

## Conclusion

Comprehensive understanding of the topoisomerase isoforms may have guiding significance for the diagnosis and prognosis in EOC patients. On the basis of our study, the discovery of the systematic molecular mechanisms that how topoisomerase isoforms reflect or lead to different outcomes of tumor patients can pave a way for more effective tumor diagnosis and treatment.

## Additional file

Additional file 1:
**Table S1.** Correlation of topoisomerase isoenzymes with chemotherapy drugs contained in therapeutic regimen. (DOCX 16 kb)
