# Supplementary material for: Targeting of topoisomerases for prognosis and drug resistance in ovarian cancer
Source: J Ovarian Res. 2016 Jun 18;9:35. doi: 10.1186/s13048-016-0244-9 (PMC4912764; doi:10.1186/s13048-016-0244-9)
Supplement: Additional file 1: — Table S1. Correlation of topoisomerase isoenzymes with chemotherapy drugs contained in therapeutic regimen. (DOCX 16 kb) [file 13048_2016_244_MOESM1_ESM.docx]

**Table S1** Correlation of topoisomerase isoenzymes with chemotherapy drugs contained in therapeutic regimen.

| Drug | Isoenzymes | Case-low | Case-high | HR (%95 CI) | *P*-value |
| --- | --- | --- | --- | --- | --- |
| Platin | TOP1A | 996 | 339 | 1.19(1.01−1.41) | 0.038 |
|  | TOP2A | 612 | 723 | 1.15 (1−1.34) | 0.054 |
|  | TOP2B | 484 | 851 | 1.13(0.97−1.32) | 0.11 |
|  | TOP3A | 989 | 346 | 0.79(0.67−0.93) | 0.0049 |
|  | TOP3B | 892 | 443 | 0.84(0.72−0.99) | 0.032 |
| Taxol | TOP1A | 551 | 201 | 1.61(1.31−1.97) | 4.8e−06 |
|  | TOP2A | 489 | 263 | 1.35(1.1−1.65) | 0.0032 |
|  | TOP2B | 493 | 259 | 1.22 (1−1.49) | 0.055 |
|  | TOP3A | 390 | 362 | 0.78(0.64−0.95) | 0.013 |
|  | TOP3B | 498 | 254 | 0.76(0.61−0.94) | 0.011 |
| Taxol +Platin | TOP1A | 534 | 201 | 1.63(1.33−2.01) | 2.7e−06 |
|  | TOP2A | 357 | 378 | 1.38(1.13−1.69) | 0.0014 |
|  | TOP2B | 479 | 256 | 1.22(0.99−1.49) | 0.057 |
|  | TOP3A | 380 | 355 | 0.78(0.64−0.95) | 0.015 |
|  | TOP3B | 233 | 502 | 0.76(0.62−0.93) | 0.0071 |
| Topotecan | TOP1A | 48 | 71 | 2.25(1.49−3.39) | 7.5e−05 |
|  | TOP2A | 29 | 90 | 1.71(1.08−2.72) | 0.021 |
|  | TOP2B | 60 | 59 | 1.26(0.87−1.84) | 0.22 |
|  | TOP3A | 71 | 48 | 1.31(0.9−1.92) | 0.16 |
|  | TOP3B | 86 | 33 | 0.72(0.47−1.11) | 0.13 |
| Paclitaxel | TOP1A | 58 | 162 | 0.71(0.43−1.17) | 0.18 |
|  | TOP2A | 93 | 127 | 0.65(0.41−1.04) | 0.07 |
|  | TOP2B | 128 | 92 | 0.65(0.41−1.03) | 0.062 |
|  | TOP3A | 158 | 62 | 0.59(0.35−1.01) | 0.053 |
|  | TOP3B | 54 | 166 | 0.7(0.44−1.12) | 0.13 |
| Docetaxl | TOP1A | 68 | 40 | 2.92(1.63−5.22) | 0.00018 |
|  | TOP2A | 75 | 33 | 2.6(1.48−4.56) | 0.00057 |
|  | TOP2B | 44 | 64 | 1.66(0.94−2.93) | 0.078 |
|  | TOP3A | 70 | 38 | 1.4(0.79−2.47) | 0.24 |
|  | TOP3B | 77 | 31 | 1.65(0.94−2.91) | 0.078 |
| Awastin | TOP1A | 36 | 14 | 2.66(1.08−6.58) | 0.027 |
|  | TOP2A | 31 | 19 | 2.41(0.93−6.28) | 0.062 |
|  | TOP2B | 36 | 14 | 2.82 (1.06−7.45) | 0.029 |
|  | TOP3A | 26 | 24 | 0.67(0.27−1.64) | 0.38 |
|  | TOP3B | 26 | 24 | 0.33(0.11−1) | 0.041 |
